# Investigating the Neural Correlates of the Attention Training Technique Using a Novel fMRI Paradigm for Measuring Attentional Control

**DOI:** 10.1002/hbm.70416

**Published:** 2025-11-24

**Authors:** Kristina Schwarz, Adrian Wells, Franziska Giller, Tanja Endrass

**Affiliations:** ^1^ Department of Clinical Psychology and Psychotherapy Technische Universität Dresden Dresden Germany; ^2^ Division of Psychology and Mental Health, School of Health Sciences, Faculty of Biology, Medicine and Health The University of Manchester Manchester UK

**Keywords:** attention training technique, attentional control, fMRI, fronto‐parietal control network, parametric modulation

## Abstract

Attentional control (AC) plays a causal role in various mental disorders and, within the metacognitive model, contributes to maladaptive repetitive cognitive processes such as rumination and worry. The Attention Training Technique (ATT), an auditory psychotherapeutic intervention, improves AC and is associated with the efficiency of large‐scale fronto‐parietal control networks (FPN). This study investigates the neural correlates of ATT by applying a newly tailored fMRI paradigm, focusing on FPN engagement and its relationship with AC. We adapted ATT to examine neural responses during ATT compared to passive listening in ATT‐naïve participants (*N* = 43) and ensured the robustness of results by validating the findings in a second independent sample (*N* = 28). To optimize the paradigm, we compared two ATT conditions, rapidly switching (ATT_switch_) and selectively focusing (ATT_focus_) attention, against multiple passive‐listening control conditions, to probe ATT‐related FPN activation. We also tested whether trial‐wise subjective effort and self/external focus ratings differentiated ATT from control trials, parametrically modulated FPN activation, and whether ATT‐related FPN activation correlated with trait AC. ATT versus control conditions activated the FPN (p_FWE_ < 0.05). This effect was present in both ATT conditions, with stronger activation in the ATT_switch_ versus ATT_focus_ condition, and independent of the specific control condition. Ratings of self/external focus and effort significantly differentiated ATT from control conditions (all *p* < 0.001) and parametrically modulated FPN activation (p_FWE_ < 0.05). All effects were replicated in the second sample. Across both samples, FPN activation in ATT versus control conditions and trial‐wise ratings related to trait AC. Using a novel fMRI paradigm in two independent samples, we demonstrate that the ATT is associated with activation of the FPN, a key network for AC and mental health. The relationship between FPN activation and self‐report measures supports the relevance of the data for understanding ATT and its links to clinical phenotypes.

## Graphical abstract

We adapted a newly tailored fMRI paradigm to assess the neural processes during the Attention Training Technique (ATT), which aims to increase the ability to disengage from perseverative thinking. Our results highlight that ATT engages the fronto‐parietal control network, with activation linked to task performance and self‐reported AC.

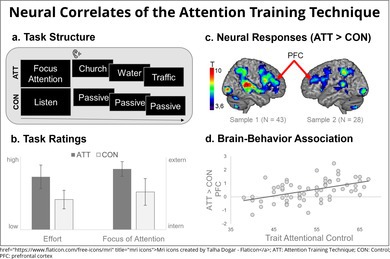

**Summary**

- We used a refined fMRI paradigm to examine the neural correlates of the Attention Training Technique (ATT)—a psychotherapy technique effective in treating negative repetitive thinking—optimizing design and behavioral assessments across two independent samples.
- ATT engagement was associated with increased activity in the fronto‐parietal control network and related to self‐reported attentional control, highlighting the neural correlates of attentional modulation.
- The paradigm offers a novel approach for examining ATT‐related neural correlates, enabling future studies to further investigate its clinical relevance.

## 1 Introduction

Attentional control (AC) is multifaceted, involving the selection and regulation of information processing intensity and breadth. Alterations in AC abilities are implicated in multiple psychological disorders such as major depression and anxiety disorders (for example Hsu et al. 2015, 2019) and linked to repetitive negative thinking such as rumination and worry (Fajkowska and Derryberry 2010; Koster et al. 2011). Psychological theories of AC differ in the proposed mechanisms, which range from structural components (e.g., network strength), capacity limitations on processing, to reflexive versus strategic control. Wells et al. considered such mechanisms in the self‐regulatory executive function (S‐REF) model, suggesting that dysfunctional AC and attention biases in psychological disorders stem from top‐down executive factors, including higher‐order knowledge and coping strategy choices, such as voluntary control over attention (Wells 2019; Wells and Matthews 1994). In particular, Wells and Matthews (1994) introduced the concept of a transdiagnostic cognitive‐attentional syndrome (CAS) which involves the prioritization of perseverative thinking (i.e., worry and rumination), threat monitoring, and unhelpful coping strategies as a common causal factor in emotional disorders. Within the S‐REF model, the CAS is regulated by a higher‐order metacognitive control system (Wells 2019), and persists due to maladaptation in executive control of attention and knowledge of control. To address these maladaptations, metacognitive therapy (MCT) (Wells 2009) employs specific therapeutic techniques aimed at improving metacognitive control. One such technique is the Attention Training Technique (ATT) (Wells 1990), which is often used within the MCT treatment package but has also been found to be beneficial as a stand‐alone intervention (Fergus and Bardeen 2016; Knowles et al. 2016).

The ATT is a complex multicomponent method that was designed to increase flexibility of attention and reduce the intensity and allocation of resources to self‐focused/internal processing that is the basis of the CAS. The procedure consists of three sets of auditory exercises that are usually combined into a single practice session. Specifically, patients are instructed to listen to an audio recording of various sounds played simultaneously and to (1) focus selectively on one single sound for a longer period of time, (2) switch rapidly between sounds, and (3) expand their awareness and try to process multiple sounds simultaneously. Repeated ATT increases AC and enhances cognitive flexibility, thereby alleviating psychological symptoms such as rumination (Wells 2009). Despite its clinical efficacy (Knowles et al. 2016), and increasing neuroscientific interest (e.g., Jahn et al. 2023; Kowalski et al. 2020; Müller et al. 2025; Rosenbaum et al. 2018), a well‐controlled fMRI paradigm to investigate the neural effects of ATT is lacking.

Cole et al. (2014) introduced a transdiagnostic neurocognitive theory paralleling the concept of the CAS and S‐REF, suggesting that cognitive control—a domain‐general pathophysiological mechanism in psychiatric conditions—is linked to the efficiency of large‐scale brain networks, such as the fronto‐parietal control network (FPN). This network includes prefrontal (lateral, dorsomedial, inferior) and parietal regions, which are highly interconnected with each other and other networks across domains and tasks (Cieslik et al. 2015; Müller et al. 2015). According to Cole et al. (2014), FPN efficiency helps buffer symptoms by enabling the use of executive control strategies, compensating for downstream abnormalities. Neuroimaging paradigms that assess AC, consistently target the FPN (Zelazo 2020) and alterations in this network have been reliably linked to psychopathological states and AC impairments across psychological disorders (Fani et al. 2019; Klumpp et al. 2018). In line, existing neuroscientific studies on ATT‐related neural correlates suggest that key hubs of the FPN are implicated in the ATT using electroencephalography (EEG) (Knowles and Wells 2018), functional magnetic resonance imaging (fMRI) (Jahn et al. 2023; Kowalski et al. 2020; Siegle et al. 2007) and functional near‐infrared spectroscopy (fNIRs) (Rosenbaum et al. 2018) Specifically, prior studies suggest that ATT can alter neural activity and functional connectivity, including reduced control network activation during emotional processing (Jahn et al. 2023) and diminished FPN connectivity during rumination (Kowalski et al. 2020) or default mode network connectivity in patients with major depressive disorder (Müller et al. 2025). Most previous work has examined changes in resting‐state activity or task performance before and after ATT training, thereby limiting direct inferences about neural processes during the actual execution of ATT. Only a few studies to date have examined the neural correlates of ATT during ATT itself, each using different methodological approaches and control conditions.

Using EEG source localization, Usui et al. (2022) examined brain activity during a short resting baseline and subsequent ATT performance. While the within‐subject design offers important insights into neural changes during ATT, the brief and uncontrolled baseline limits interpretability. In contrast Kowalski et al. (2020) proposed a promising between‐subjects approach by comparing brain synchronicity between a group undergoing a naturalistic ATT session and a group listening to the identical sounds without ATT instructions—and found ATT‐related increased synchrony in large‐scale control networks, including the FPN. Yet, the between‐group design remains susceptible to interindividual variability and pre‐existing trait differences, complicating the attribution of effects to the intervention itself. To address such limitations, Rosenbaum et al. (2018) developed a neuroscience‐compatible ATT paradigm for fNIRS, allowing greater experimental control by contrasting short ATT intervals with a white‐noise passive‐listening control condition. Their findings replicated increased FPN activation during ATT. However, this control condition lacks comparable perceptual demands, making it difficult to isolate neural activity specifically related to AC processes elicited by ATT.

In the present study, we adapted the paradigm by Rosenbaum et al. (2018) for fMRI and piloted it in two independent samples of healthy participants. We focused on two core components of the ATT: selective focusing (ATT_focus_) and rapid switching (ATT_switch_) to reduce task complexity, and ensure sufficient trial numbers. Alongside the white noise passive listening control condition (Rosenbaum et al. 2018), we included two higher‐level passive‐listening control conditions to balance auditory complexity. In psychotherapy, ATT effectiveness is assessed through a self‐attention rating which acts as a surface marker of CAS activity, where successful practice is indicated by a reduction in self‐focused attention (Wells 1990). We similarly collected trial‐wise self‐attention (i.e., internal vs. external focus) and subjective effort ratings (Rosenbaum et al. 2018). We predicted that a more external focus and higher effort in ATT trials would be evident when compared to control trials. To ensure the robustness of results, we replicated the findings in a second independent sample. Further, we explored associations between brain activity in the FPN during ATT and behavioral measures, including self‐attention and effort ratings. Finally, we examined relationships with self‐reported individual differences in AC, assessed by the Attentional Control Scale (ACS, Derryberry and Reed 2002).

## 2 Methods and Materials

### 2.1 Sample

In total, 71 healthy individuals participated in this study. These participants were recruited sequentially in two separate samples. After completing data collection in the first sample, a few modifications were made to the ATT task (see details in the ATT paradigm section below) before recruiting and testing the second sample (see Table 1). Participants were recruited via public advertisement. Inclusion was limited to individuals without current psychological disorders, as assessed by a structured telephone interview using the SCID‐5 screening module. None of the participants reported current psychotherapeutic treatment or the use of psychoactive medication. In addition, we assessed the CAS‐1, a 16‐item self‐report questionnaire measuring maladaptive thinking patterns and attentional strategies (e.g., worry, rumination, threat monitoring). While Kowalski et al. measured the CAS‐1 in a large sample and recruited extreme groups on CAS measures, our samples were not selected by CAS and show typical, non‐extreme mean scores (see Table 1; Kowalski et al. 2019; Kowalski et al. 2020; Kowalski and Dragan 2019). Additional exclusion criteria were (1) prior experience with ATT training, (2) fMRI contraindications, (3) past or present neurological disorders, cardiovascular disease, or head trauma, and (4) hearing disabilities. All participants provided written informed consent, and the procedures were approved by the local Ethics Committee (SR‐EK‐151032023).

**TABLE 1:** Sociodemographic characteristics.

	Sample 1 (*N* = 43)		Sample 2 (*N* = 28)	
Sex (male/female)	16/27		9/19	

	Mean	SD	Mean	SD
Age	27.30	7.62	26.14	6.77
Effort Rating				
ATT	3.54	0.93	3.70	0.50
CON	2.05	0.71	2.06	0.55
External/Self Focus Rating				
ATT	4.13	0.47	4.14	0.55
CON	2.44	0.85	2.79	0.98
ACS	53.10	8.21	53.39	5.92
CAS	57.54	14.89	59.10	12.86

*Note:* Effort Ratings are scaled 1 (low effort) to 5 (high effort). Attention Ratings are scaled 1 (internal) to 5 (external).

Abbreviations: SD: standard deviation, ACS: Attentional Control Scale (Derryberry and Reed 2002), CAS: Cognitive Attentional Syndrome Scale.

### 2.2 ATT Paradigm

**FIGURE 1:**
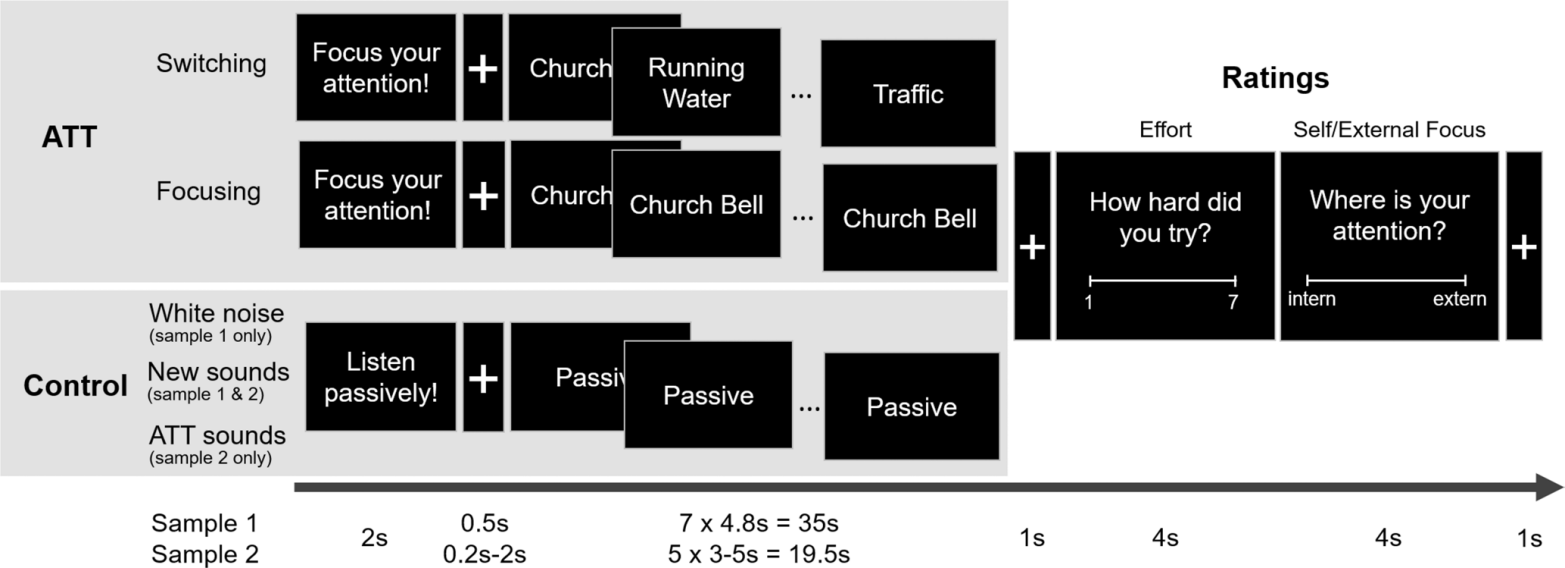
ATT Paradigm (adapted from Rosenbaum et al. 2018). ATT: Attention Training Technique condition; CON: Control condition; ATT Switching: Rapidly switch the attentional focus between sounds; ATT Focusing: Focus attention on a specific sound for a longer period of time; Control White noise: Passively listen to white noise (low‐level control); Control New Sounds: Passively listen to alternative sounds; Control ATT sound: Passively listen to ATT sounds. s: Seconds.

We developed an fMRI adapted version of the ATT paradigm used by Rosenbaum et al. (2018) and tested it in to two independent samples of healthy individuals (see Figure 1). Participants listened to standardized ATT sounds (including sounds of clock ticking, church bell, bird song and traffic) previously used in ATT research available at http://www.metakognitivetherapie.de/, see also (Barth et al. 2019; Rosenbaum et al. 2018) or to control sounds (see Figure 1 and Supporting Information for stimulus details). Sounds were presented using high‐quality noise‐cancellation headphones optimized for use in the MRI environment (https://www.optoacoustics.com/medical/optoactive/features), ensuring consistent and clear auditory presentation.

The clinical ATT typically involves a continuous audio stream lasting about 12 min including three components: 6 min of sustained selective attention (focusing on one sound), 4 min of rapid switching between sounds, and 2 min of divided attention (attending to multiple sounds simultaneously). In line with Rosenbaum et al. (2018), our paradigm adapts this structure to neuroimaging constraints by using discrete, clearly separated blocks. Because the divided attention condition accounts for the shortest portion of the ATT training and in order to reduce task complexity, and ensure sufficient trial numbers, we omitted the divided attention condition and focused on rapid switching (ATT_switch_) and selective focusing (ATT_focus_). ATT trials were contrasted with control conditions involving passive listening to alternative sounds (CON_sounds_) and white noise (CON_white_, sample 1) or passive listening to the ATT sounds (CON_ATT_, sample 2) to better isolate ATT effects.

To assess task engagement and attention allocation, participants rated (1) the direction of their current attentional focus (internal vs. external) and (2) how hard they tried to fulfil the task (low to high effort; see Figure 1). Each condition was presented four times (6 times in sample 2) in a pseudorandomized order. In sample 2, trials were shortened to 20 s (compared to 35 s) in order to increase the number of trials without exceeding task duration, resembling the recommended training time in the MCT (Wells 2009) and the recommended duration used in fMRI block designs (Maus et al. 2010). For a detailed overview of task design choices, trial structure, timing and procedure, see Supporting Information.

### 2.3 fMRI Parameters and Preprocessing

Functional (TR: 869 ms, TE: 38 ms, flip angle 58°, FOV: 264 mm, and 60 axial slices, voxel size: 2.4 mm^3^) and structural (TR: 2000 ms, TE: 1.97 ms, flip angle 8°, FOV: 256 mm, 208 slices, voxel size: 1 mm^3^) images were obtained on a 3 Tesla Trio Siemens scanner. Standard preprocessing was performed in SPM12 (http://www.fil.ion.ucl.ac.uk/spm/), including slice‐time correction, realignment to the mean image using field map correction, indirect normalization and spatial smoothing (8 mm). Participants with a mean frame‐wise displacement (Power et al. 2012) greater than 0.5 mm across all frames and/or in over 20% of all acquired frames were excluded from further analyses (sample 1: *N* = 2; sample 2: *N* = 2) resulting in the final sample of 71 participants (sample 1: *N* = 43; sample 2: *N* = 28; see Table 1). Overall, significance was assessed at the voxel level and defined a priori as *p* < 0.05, family‐wise error (FWE) corrected within the whole brain and within a combined mask comprising the prefrontal parts of the right and left FPN as defined by Glasser et al. (2016); see Supporting Information for details. We used the Glasser atlas to distinguish the FPN from language networks, which both involve prefrontal cortex regions. This is important given that, although ATT conditions are similar in structure and word count, the switching condition's changing words likely increase demands on semantic and linguistic processing, potentially activating language‐related regions in addition to the FPN. The ROI mask, scripts, and main data that support the findings of this study are available in the Open Science Framework (OSF) at https://osf.io/scbrj/.

### 2.4 Functional Activation of the ATT

At the first level, task regressors for the ATT conditions (ATT_switch_ and ATT_focus_) as well as the used control conditions (CON_sounds_, CON_white_, CON_ATT_) were entered into a general linear model (GLM) together with regressors for the instruction and self/external focus as well as effort ratings and the six motion parameters as nuisance regressors. To investigate the functional activation during ATT compared to control conditions, individual contrast estimates for ATT>CON were subjected to a second‐level one‐sample t‐test. Further second‐level analyses were performed to better understand the neural responses of the different task conditions. Specifically, we tested for differential effects of (1) the two ATT conditions (ATT_switch_ vs. ATT_focus_) and (2) the three different control conditions (see Table S1 for the investigated contrasts of interest). Analyses were done separately in both samples in order to replicate the results and ensure their robustness across different datasets. Given the differences in task timing, we explored differences in the ATT>CON_sound_ contrasts between samples using a two‐sample *t*‐test. Additionally, to assess whether increased linguistic demands in the ATT_switch_ condition contributed to differences from the ATT_focus_ condition, we examined the overlap of activation patterns with the Glasser‐defined language network (see Figure S2).

### 2.5 Attention and Effort Ratings and Parametric Modulation Analyses

As behavioral performance in the ATT cannot be assessed, trial‐wise self/external focus of attention and effort ratings were collected. To test whether the ratings differentiate between ATT and CON trials, we performed repeated measures analyses of variance in SPSS (IBM, SPSS, version 29) including the mean scale rating for each task condition for self/external focus and effort ratings, respectively. In a second step, we conducted parametric modulation analyses to assess whether subjective experiences of attentional focus and mental effort were reflected in neural activity during the ATT task. Trial‐wise self−/external‐focus and effort ratings were entered as parametric modulators instead of the task regressors. That is, the models did not contain separate regressors for ATT and CON conditions; rather, they tested whether brain activity covaried with the continuous rating values across trials. This approach follows the method described by Büchel et al. (1998), which is a well‐established method to model continuous trial‐wise predictors to explain variation in neural activation within experimental conditions. This approach allowed us to examine whether subjective effort or attentional orientation was associated with activation of FPN regions typically engaged during AC. First‐level contrast images of the parametric modulators were entered into second‐level one‐sample *t*‐tests. Analyses were conducted separately for each sample to test the robustness of findings.

### 2.6 Neural and Behavioral Associations With AC

Trait AC was assessed using the ACS (Derryberry and Reed 2002), a 20‐item questionnaire that includes a total score for AC and multiple studies reported two‐factor solutions comprising the subscales of focusing (9 items) and shifting (11 items) (Judah et al. 2014; Ólafsson et al. 2011). Total scores range from 20 to 80, with higher scores indicating higher AC. The scale has adequate psychometric properties (Fajkowska and Derryberry 2010). Unfortunately, questionnaire data were not available for 3 subjects, leaving *N* = 68 datasets in the following analyses. To increase statistical power, we combined both samples for the analyses. As the task design between the two samples was slightly different, we included task version as a covariate of no interest.

First, we investigated whether self‐reported AC was associated with self/external focus and effort scores in the ATT task by adding sum scores of the ACS as a covariate to the repeated measures analyses described above, while controlling for task version. Second, to probe the brain‐behavior association between ATT‐related brain responses in the FPN and trait AC, the sum scores of the ACS were included as an additional regressor in second‐level analyses, respectively. We chose to combine ATT conditions, as we expect them to similarly recruit the FPN (Rosenbaum et al. 2018). For the control condition, we chose CON_sound_, because the condition was identical in both samples (for more details see task description).

## 3 Results

### 3.1 Functional Activation of the ATT

**FIGURE 2:**
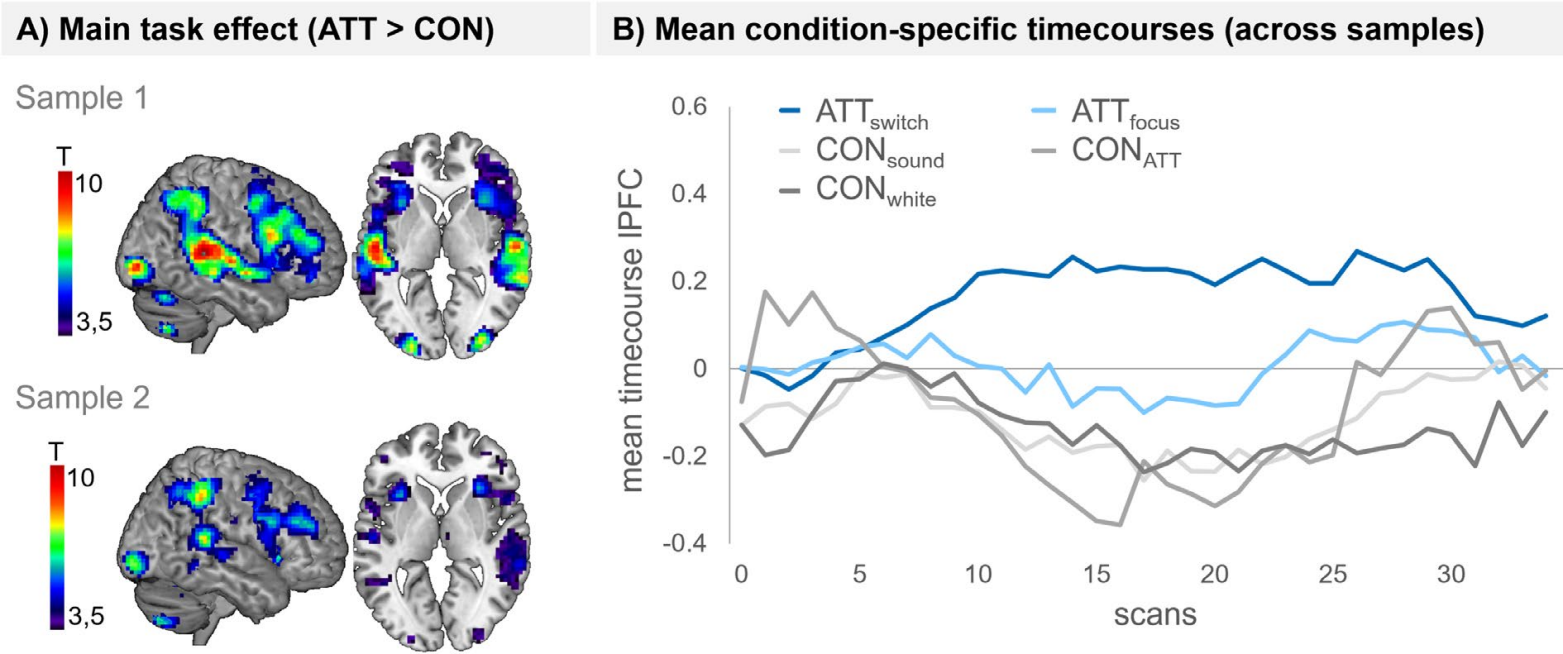
Main effect of Task. (A) Overall results of the one‐sample *t*‐tests contrasting ATT (rapid switching and focusing) versus Control (CON) conditions, displayed for sample 1 and sample 2, respectively. Maps were thresholded at the critical *T*‐value of 3.67 (*p* < 0.05), using small‐volume correction for the prefrontal part of the fronto‐parietal network as defined by Glasser et al. (2016). Note the differences in statistical power between sample 1 (*N* = 43) and sample 2 (N = 28). (B) Mean time courses in the lateral prefrontal cortex (lPFC) region of interest for each condition across samples. CON_white_: White noise; CON_sound_: Alternative sounds; CON_ATT_: Sounds used in ATT condition.

ATT versus CON: Whole brain analyses revealed that ATT compared to control conditions led to higher activation in the FPN, including the bilateral PFC and intraparietal sulcus, but also in the superior temporal, occipital gyrus and cerebellum (p_FWE_ < 0.05; see Figure 2 and Table S2). ROI analyses confirmed the engagement of the lateral PFC (see Table S3). Results were replicated in the second independent sample.

**FIGURE 3:**
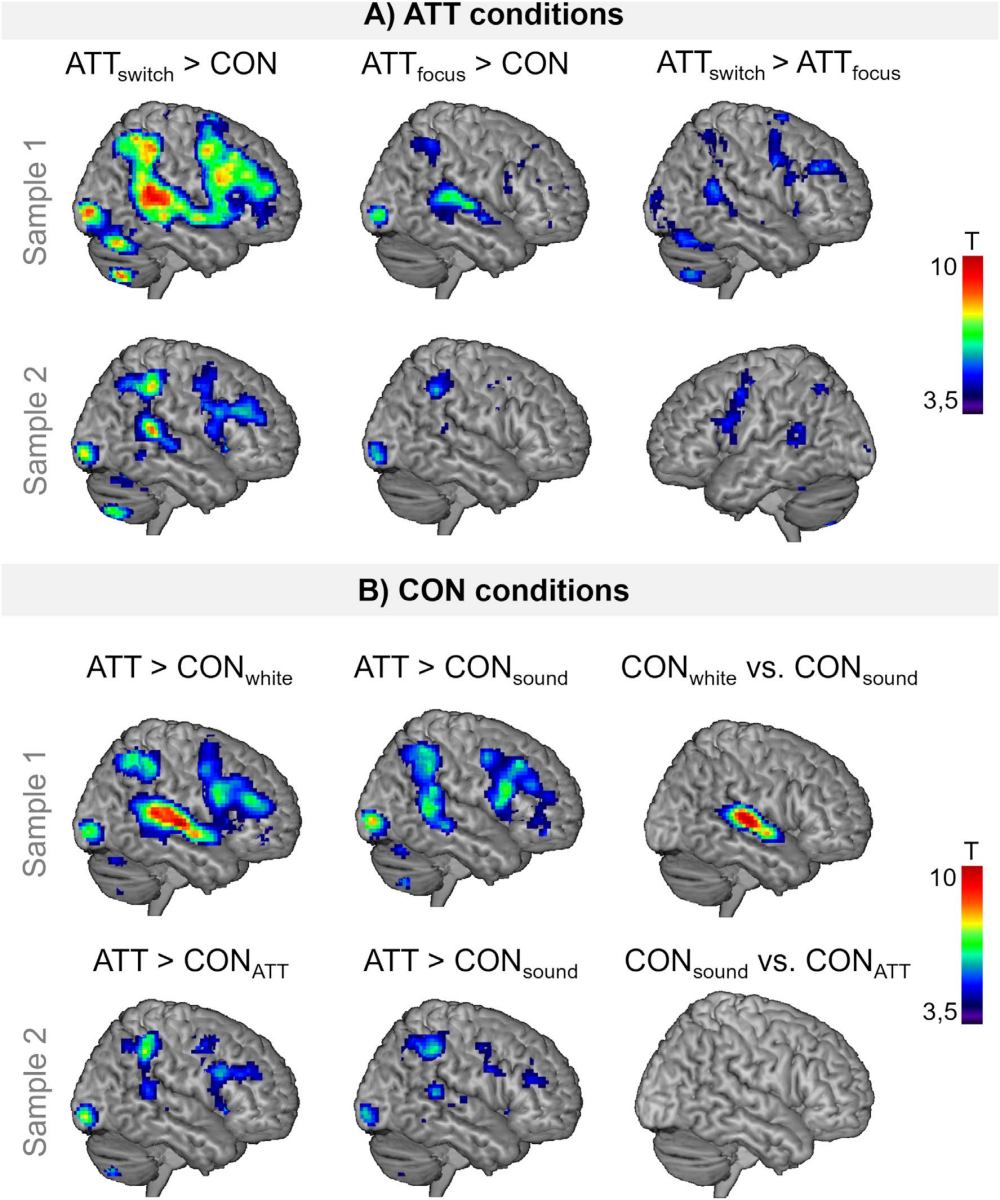
Differential neural activation during ATT versus control (CON) conditions. (A) Comparison of the differential effects of ATT conditions (rapid switching versus focusing) displayed for sample 1 and sample 2, respectively. (B) Differential effects of passive‐listening control conditions with varying sound input (CON_white_: White noise; CON_sound_: Alternative sounds; CON_ATT_: Sounds used in ATT condition). For illustration purposes, a significance threshold of *p*_uncorrected_ < 0.005 was applied. Note the differences in statistical power between sample 1 (*N* = 43) and sample 2 (*N* = 28).

Differential effects of ATT conditions: Compared to control conditions, both ATT conditions (ATT_switch_ and ATT_focus_) activated the FPN (*p*_*FWE*_ < 0.05) in whole brain analyses, although task effects were stronger in the ATT_switch_ versus ATT_focus_ condition as revealed by the direct comparison between conditions (see Figure 3A and Table S4 for whole brain results). Other networks beyond the FPN included temporal, occipital, cerebellar regions, and—in the second sample—the bilateral insula. ROI analyses confirmed the engagement of the lateral PFC for both ATT conditions across both samples (see Table S5). Exploratory control analyses show robust activations in the superior temporal cortex and part of the inferior frontal cortex, overlapping with the language network, but also distinct clusters in parietal and prefrontal regions, specifically overlapping with the FPN (see Figure S2).

Differential effects of control conditions: Larger neural activation in the FPN was observed in ATT compared to all control conditions (CON_sound_, CON_white_, and CON_ATT_, all p_FWE_ < 0.05 respectively, see Figure 3B and Table S6). The strongest effects were observed comparing ATT to the CON_white_ condition where effects in whole brain analyses were more widespread and strongly activated the auditory cortex, as also seen when contrasting CON_white_ to CON_sound_ (see Figure 3B). No differences between the higher‐level passive listening CON_sound_ and CON_ATT_ conditions emerged, and the contrast between ATT and these control conditions revealed more specific FPN activation (see Figure 3B). ROI analyses confirmed the engagement of the lateral PFC when comparing ATT to any of the used control conditions (see Table S7).

Differences between task version: Exploratory analyses revealed no significant activation differences between the two task versions, neither at the whole‐brain level nor within the ROI (all *p*_*FWE*_ > 0.05).

### 3.2 Trial‐Wise Self/External Focus and Effort Ratings and Parametric Modulation Analyses

**FIGURE 4:**
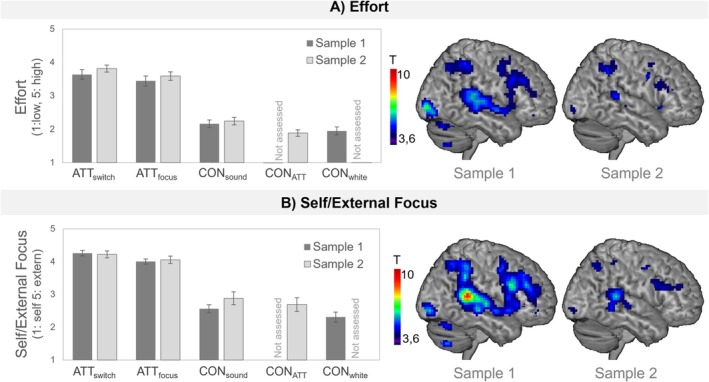
Behavioral and neural effects of trial‐wise Effort/Attention Scales. Left side: Bar plots depict differences in effort (A) and self/external focus (B) ratings (mean/standard error) for sample 1 and 2, respectively. CON: Control; CON_white_: White noise; CON_sound_: Alternative sounds; CON_ATT_: Sounds used in ATT condition. Right side: Effects of parametric modulation analyses including trial‐wise effort (A) and self/external focus (B) ratings as parametric modulators in individual first‐level analyses in sample 1 and sample 2, respectively. Maps were thresholded at the critical *T*‐value of 3.67 (*p* < 0.05, small‐volume corrected for the prefrontal part of the fronto‐parietal network as defined by Glasser et al. (2016)).

Effort and Self/External focus ratings: Trial‐wise effort and self/external focus ratings significantly differed between conditions, respectively (effort: *F*_(1.4,58.8)_ = 54.91, *p* < 0.001, *η* ^*2*^ = 0.57; self/external focus: *F*_(1.8,74.0)_ = 113.90, *p* < 0.001, *η* ^*2*^ = 0.73). This was replicated in the second sample (effort: *F*_(1.6,42.9)=_75.44, *p* < 0.001 *η* ^*2*^ = 0.73; self/external focus: *F*_(1.7,45.9)_ = 30.94, *p* < 0.001 *η* ^*2*^ = 0.53; see Figure 4). Post hoc *t*‐tests revealed that subjects spent more effort, and had a more external attentional focus in both ATT conditions compared to control conditions (all *p < 0*.001), with large effect sizes (*d*_*z*_ ranged 1.05–2.14; for details see Figure 4 and Table S8). Specifically, effort ratings and external focus ratings were higher in the ATT_switch_ compared to the ATT_focus_ conditions with moderate effect sizes in both samples (effort: *d*_*z*_≈0.40–0.43; self/external focus: *d*_*z*_≈0.41–0.55), although significance was reached only in sample 1 after Bonferroni‐Holm correction, likely due to reduced statistical power in the smaller sample 2. Mean scale ratings differed between control conditions (all *p* < 0.05), with small to moderate effect sizes (*d*_*z*_ ranging 0.22–0.72), except for self/external focus ratings, which did not differ significantly between CON_ATT_ compared to CON_sound_ conditions in sample two (*p* = 0.25, *d*_*z*_ = 0.22).

Parametric Modulation analyses: Including the ratings as parametric modulators in the first‐level analyses led to similar activation patterns compared to the task effect (ATT>CON) in both samples. Specifically, ROI analyses showed that effort and self/external focus ratings were related to neural responses in the lateral PFC, respectively (p_FWE_ < 0.05; see Figure 4, Table S9–S10).

### 3.3 Neural and Behavioral Associations With AC

**FIGURE 5:**
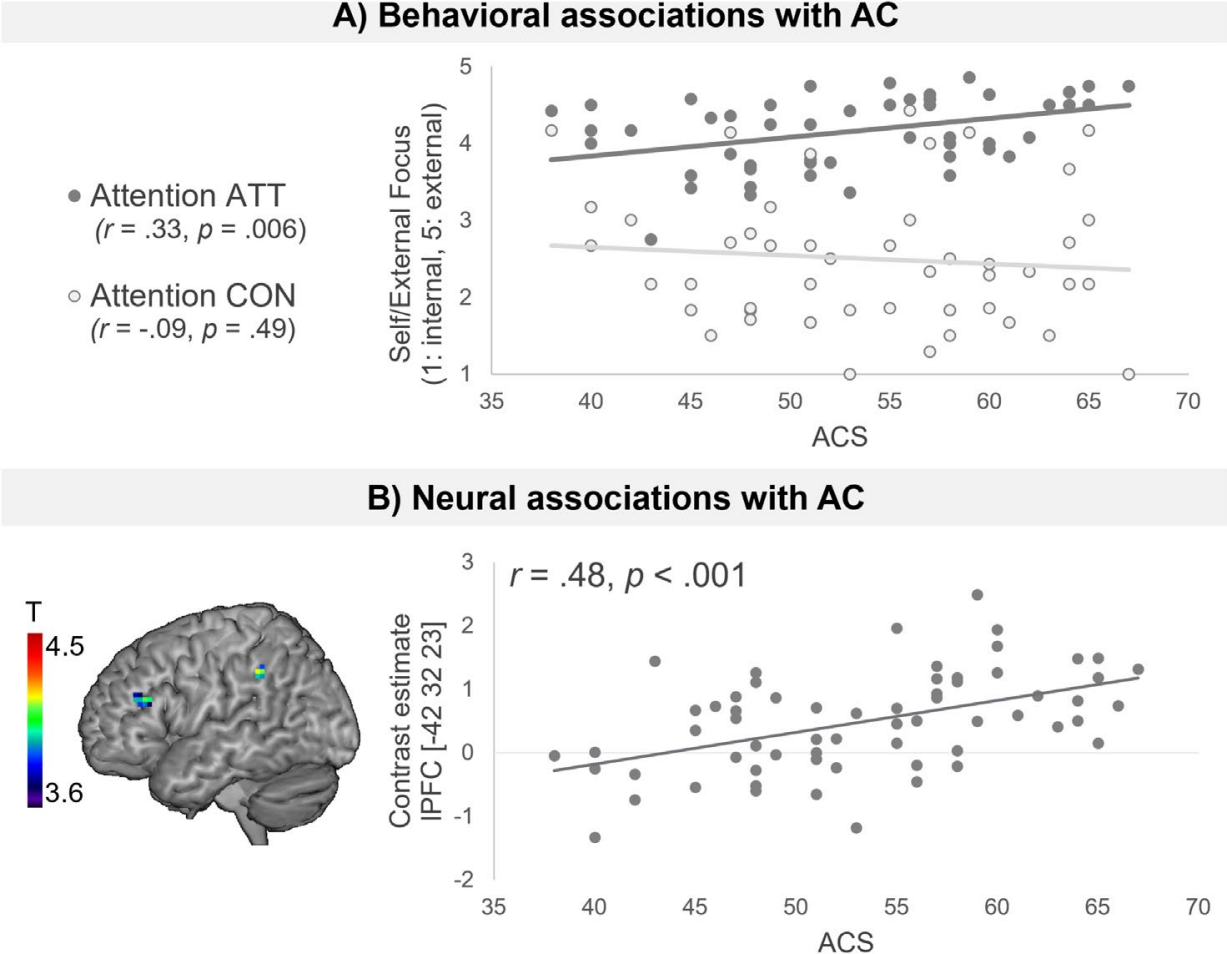
Behavioral and neural associations with trait‐AC. Associations of self‐reported Attentional Control (AC), assessed using total scores of the Attentional Control Scale (ACS; Derryberry and Reed 2002) and behavioral (A) as well as neural (B) task effects. CON: Control (combined across control conditions); ATT: Attention Training conditions (combined across ATT conditions). lPFC: Lateral prefrontal cortex. Maps were thresholded at the critical *T*‐value of 3.67 (*p* < 0.05, small‐volume corrected for the prefrontal part of the fronto‐parietal network as defined by Glasser et al. (2016)).

Behavioral level: A significant interaction effect between self/external focus ratings and self‐reported AC (*F*_(*1,65*)_ = 4.489, *p* = 0.038, partial *η* ^*2*^ = 0.065) was observed. Specifically, self‐report AC related to a more external attention direction in ATT while being unrelated in control trials (see Figure 5A). In contrast, no interaction effects were observed for self‐reported AC and effort ratings (*F*_(1,65)_ = 1.540, *p* = 0.219, partial *η* ^*2*^ = 0.023).

Neural level: Similarly, self‐report AC related to neural activation in the left lateral PFC ROI (see Figure 5B, Table S11), while no associations emerged with effort ratings.

## 4 Discussion

This study offers valuable insights into the neural correlates of the ATT—an auditory psychotherapeutic technique designed to enhance metacognitive control over processing by increasing the ability to disengage from perseverative, self‐focused thinking, such as worry and rumination (Wells 2009). We developed an fMRI paradigm to investigate brain responses during ATT compared to passive listening and demonstrate that its core elements (i.e., focus attention and switching attention) recruit the FPN, pivotal for cognitive control. Our study replicates previous findings (Rosenbaum et al. 2018) and extends this work by: (1) adapting the paradigm for fMRI, (2) enhancing its efficiency through optimized task design, scale ratings, and the development of parallel control conditions, (3) replicating task effects in a second independent sample, and (4) demonstrating the validity of ATT‐related brain responses in capturing AC by showing associations between FPN activation and self‐reported trait AC.

### 4.1 FPN Engagement During ATT

As shown independently in both samples, performing the ATT reliably engaged the FPN, involving parts of the inferior and lateral PFC as well as parietal cortices. While this finding is consistent with Rosenbaum et al. (2018), who reported increased FPN activation during a neuroscience‐adapted ATT task using fNIRS, our paradigm extends these findings by employing fMRI and incorporating more specific control conditions. Our findings further align with a range of neuroscientific evidence demonstrating FPN involvement in ATT‐related processes. This has been shown across methodologies (EEG, fMRI, fNIRS) and experimental contexts—ranging from resting‐state assessments (Knowles and Wells 2018) and task‐based paradigms (Jahn et al. 2023; Siegle et al. 2007) to measurements capturing neural activity during ATT execution itself (Kowalski et al. 2020; Rosenbaum et al. 2018; Usui et al. 2022). More broadly, our findings align with literature demonstrating that auditory attention reliably recruits the FPN (Lee et al. 2014; Noyce et al. 2023; Razzaghipour et al. 2024), particularly within the PFC (Altmann et al. 2008; Bidet‐Caulet et al. 2010; Huang et al. 2012; Salmi et al. 2009). The PFC plays a central role in suppressing irrelevant auditory stimuli (Caporello Bluvas and Gentner 2013), a core requirement of the ATT. Moreover, meta‐analytic evidence indicates strong overlap between ATT‐related brain activation with supervisory control networks involving lateral PFC, parietal cortex, and anterior insula (Cieslik et al. 2015; Zhang et al. 2017). Notably, our findings show that ATT‐related brain activation is not restricted to the FPN and extends to brain networks including the dorsal attention network, visual areas, and the cerebellum. In line, Kowalski et al. (2020) found that ATT increases neural synchrony in multiple networks. However, unlike Kowalski et al. (2020), who also reported increased DMN synchrony using inter‐subject correlation, our within‐subject ATT–control contrast did not reveal DMN activation, very likely because higher synchrony does not necessarily imply increased activity and thus may not be detected in activation‐based analyses such as the ATT–control contrast. Taken together, our results support the notion that the ATT is a cognitively complex task involving multiple, interacting large‐scale brain networks centrally involving the FPN (Kowalski et al. 2020; Rosenbaum et al. 2018).

### 4.2 Neural Correlates of ATT Conditions

In addition to shared recruitment of these large‐scale networks, we also observed differential neural responses between ATT conditions (i.e., ATT_switch_ and ATT_focus_). ATT_switch_ elicited stronger responses in the FPN and extended regions, possibly reflecting higher demands on executive and linguistic‐semantic processing due to rapid word changes. Such perceptual processing has previously been linked to occipital‐temporal, fusiform, and inferior frontal areas (Murphy et al. 2019), although our data did not show the typical left‐lateralization observed in language processing. According to the Glasser functional network atlas (2016), the differential activation between ATT_switch_ and ATT_focus_ components partly overlaps with language‐related areas but also includes distinct clusters in parietal and prefrontal regions associated with the FPN. Given the similarity in perceptual processes between ATT_focus_ and control conditions—which also engage the FPN—it is unlikely that perceptual differences alone explain the FPN responses during ATT. Instead, both ATT conditions appear to rely heavily on the ability to switch attentional focus either externally guided with ATT instruction (i.e., in the ATT_switch_ condition) or self‐paced when noticing attention drifts (i.e., in the ATT_focus_ condition; Rosenbaum et al. 2018). Overall, our results suggest that attention focusing and switching engage overlapping brain networks, with the FPN playing a crucial role. This is consistent with previous ATT research (Rosenbaum et al. 2018), aligns with recent models of (auditory) attention (Razzaghipour et al. 2024), and supports the hypothesis of a shared, domain‐general AC component (Friedman and Robbins 2021).

### 4.3 Designing Effective Control Conditions

Both ATT conditions require sustained focus on specific auditory stimuli within a continuous sound stream, suppression of irrelevant input, and reorientation of attention when it drifts or needs to shift. Designing a control condition that closely matches the experimental conditions in sensory features while avoiding engagement of AC, is particularly challenging. Previous studies have addressed this by comparing neural processes during ATT either with resting‐state or passive listening conditions (Rosenbaum et al. 2018; Usui et al. 2022), or with a control group exposed to identical auditory stimuli without attentional training instructions (Kowalski et al. 2020). While the latter approach perfectly controls for auditory input, the between‐subject design is vulnerable to individual differences and trait‐related confounds. Existing within‐subject designs, on the other hand, often did not match ATT and control conditions closely enough, limiting their ability to isolate AC‐specific neural effects.

To address this problem, we tested three different control conditions with identical instructions, but varying complexity of the auditory input, more closely matching the experimental conditions. At the simplest level, consistent with Rosenbaum et al. (2018), white noise served as a control sound, producing strong brain activation in the superior temporal gyrus, likely reflecting qualitative differences in the auditory input (see Figure S1). More specific control network activation emerged when ATT trials were contrasted with higher‐level control conditions that presented either alternative sounds or the identical ATT stimuli. Notably, even with identical auditory input, active ATT performance versus passive listening elicited increased activation in the superior temporal gyrus, consistent with previous findings demonstrating that auditory attention enhances brain responses within the auditory cortex (for example Grady et al. 1997; Hall et al. 2000; Jäncke et al. 1999; Lee et al. 2014; Petkov et al. 2004; Rinne et al. 2005; Woods et al. 2009). Contrary to our expectation that participants might continue to focus attention as in the ATT condition, the two high‐level control conditions did not differ. This suggests that participants were equally able to switch attentional engagement on and off depending on the instruction, even with identical auditory input. Our findings suggest that closely matched, higher‐order control conditions can effectively account for auditory processing while isolating the targeted AC component.

### 4.4 Behavioral Correlates of the ATT Paradigm: Attention and Effort Ratings

Validating the ATT‐related activations with behaviorally relevant aspects of AC is crucial, given that the ATT itself currently lacks known directly measurable behavioral output. Like Rosenbaum et al. (2018), trial‐wise effort ratings revealed higher effort for the ATT compared to the control conditions in both samples. Participants also consistently reported a more external focus of attention after ATT compared to control trials, as measured by a rating scale that is used in MCT to measure the success of the ATT. These findings are in line with the ATT's goal of shifting attention from internal (e.g., CAS activity of sustained rumination) to external events (Wells 2009). In contrast, Kowalski et al. (2020) assessed attentional focus only before and after the entire ATT session and found no significant changes, suggesting that fine‐grained, trial‐level measures may be more sensitive to ATT effects than pre–post designs.

Effort and self/external focus ratings parametrically modulated FPN activation across both samples, supporting its role in effortful, external AC. This is in line with the S‐REF model (Wells and Matthews 1996) and previous research that has linked effort ratings in a similar paradigm to inferior frontal gyrus activation (Rosenbaum et al. 2018), which is part of the cognitive control network. Our whole‐brain approach demonstrates that both rating scales modulate brain networks similar to overall task effects. These results indicate that the ratings capture task‐relevant variance in the neural signal during the ATT, highlighting their potential as a behavioral marker for the ATT paradigm and their continued use in MCT. Nonetheless, the absence of direct behavioral measures remains a significant limitation of the task (Rosenbaum et al. 2018). Future research should explore objective markers of AC performance during the ATT, such as pupil dilation (Zhao et al. 2019) or integrating a behavioral task. Our findings still demonstrate that FPN activation aligns closely with participants' reported engagement and attentional focus, strongly indicating that participants performed the task as intended.

### 4.5 Neural and Behavioral Associations With Self‐Reported Attentional Control

The validity of the behavioral ratings is further supported by their association with trait AC. As expected, higher trait AC correlated with a greater external focus of attention during ATT trials, suggesting that individuals with higher trait AC are better able to disengage from internal distractions and focus on external stimuli during the ATT. Brain‐behavior associations further support this, with self‐reported AC abilities linked to PFC responses during ATT trials compared to control trials. These findings align with previous studies linking self‐reported AC to both behavioral performance (Derryberry and Reed 2002) and neural markers of AC (Klumpp et al. 2018), thus supporting the role of AC in cognitive flexibility and attention shifting (Hopfinger and Slotnick 2020).

Similar neural activation patterns have been reported by Kowalski et al. (2020), who found differences in lateral PFC synchrony between individuals with low and high levels of the CAS. The ACS is a trait measure capturing variability in self‐reported AC in both healthy and clinical populations, whereas the CAS primarily reflects maladaptive cognitive processes (e.g., worry, rumination, threat monitoring) typically elevated in clinical groups. According to the MCT model (Wells 2009), higher AC ability is associated with lower engagement in these maladaptive processes. In our study, participants showed moderate CAS levels; consequently, CAS‐related associations must in future be tested in samples with more elevated CAS. Supporting this, prior studies in (sub)clinical populations found neural changes following ATT interventions, including altered resting‐state connectivity (Müller et al. 2025) and task‐based activation changes (Siegle et al. 2007). Together, these findings underscore the potential of the present paradigm to detect ATT‐related neural correlates and to serve as a tool for investigating CAS effects and treatment outcomes in populations with heightened maladaptive cognitive processes.

### 4.6 Limitations

This study has several limitations. First, the paradigm deviated from the original ATT by using discrete, randomized blocks instead of a continuous auditory stream and by omitting the divided attention condition. The latter was excluded due to its short duration in the original training (Wells 2009) and earlier evidence suggesting only minor differences in brain responses (Rosenbaum et al. 2018). Nonetheless, as some studies point to a notable role of this condition (Kowalski et al. 2020; Usui et al. 2022), future research should examine ATT components more systematically using within‐subject designs. Second, designing well‐matched control conditions remains methodologically challenging, especially for auditory fMRI paradigms. The instruction in the control condition to “listen passively” might still engage low‐level attentional or inhibitory processes (i.e., exerting AC to not focus on specific sounds) and could thus be regarded as a minimally active rather than a purely passive control condition. Nonetheless, consistent with previous neuroscientific work (Jahn et al. 2023; Knowles and Wells 2018; Kowalski et al. 2019; Kowalski et al. 2020; Rosenbaum et al. 2018; Siegle et al. 2007), the ATT paradigm elicited the expected FPN activation, supporting its suitability for investigating the neurophysiological correlates of ATT in future research. Third, the absence of an objective behavioral measure of cognitive flexibility limits validation of the self‐reported attentional flexibility and interpretation of the switching condition. Future studies should integrate behavioral indices of cognitive flexibility to further elucidate underlying mechanisms. Fourth, while our sample size was modest, it is comparable to previous studies (e.g., Kowalski et al. 2020; Rosenbaum et al. 2018), and replication in an independent sample strengthens confidence in our findings. Fifth, the use of a non‐clinical, predominantly highly educated sample, limits generalization to clinical populations. Finally, findings are correlational, as causal inferences require interventional or longitudinal designs (Kazdin 2007). While previous studies have observed ATT‐related neural changes in high‐CAS and clinical samples (for example Kowalski et al. 2020; Müller et al. 2025; Rosenbaum et al. 2018; Siegle et al. 2007), applying the current paradigm in clinical contexts could help to clarify its mechanisms and inform targeted interventions.

## 5 Conclusion

This study elucidates the neural correlates of the ATT (Wells 1990), a metacognitive therapy technique targeting repetitive negative thinking common across psychological disorders. Using a newly adapted fMRI paradigm replicated in two independent samples, we provide robust evidence that the ATT effectively engages the FPN, a critical neural network for AC and mental health (Cole et al. 2014). Our results underscore important considerations for task design, such as including various control conditions and behavioral assessments. The associations between FPN activation and self‐report AC suggest that ATT‐related neural responses contribute to enhanced cognitive flexibility, which is consistent with the aims of the technique and considered critical for improving mental health outcomes in clinical settings (Wells 2009). The paradigm offers a tool to investigate ATT‐related FPN activation in relation to clinically relevant phenotypes, such as rumination, disorder‐specific symptoms, and changes following training in clinical populations. A better understanding of ATT's neural correlates may help guide precision‐based interventions targeting AC.

## Disclosure

The authors have nothing to report.

## Ethics Statement

All participants provided written informed consent, and the procedures were approved by the local Ethics Committee (SR‐EK‐151032023).

## Supporting information


**Data S1:** hbm70416‐sup‐0001‐Supinfo.docx.

## Data Availability

The data that support the findings of this study are available in the Open Science Framework (OSF) at https://osf.io/scbrj/.
