# Supplementary material for: Investigating the Neural Correlates of the Attention Training Technique Using a Novel fMRI Paradigm for Measuring Attentional Control
Source: Hum Brain Mapp. 2025 Nov 24;46(17):e70416. doi: 10.1002/hbm.70416 (PMC12644246; doi:10.1002/hbm.70416)
Supplement: Supplementary file 1 — Data S1: hbm70416‐sup‐0001‐Supinfo.docx. [file HBM-46-e70416-s001.docx]

**Supplementary information**

**Investigating the Neural Correlates of the Attention Training Technique using a Novel fMRI Paradigm for measuring Attentional Control**

1. **Task construction**

We sticked closely to the paradigm outlined in the fNRIS study of Rosenbaum et al. (2018). The paradigm consisted of two conditions:

- - - 1. ATT condition: We used a standardized German audio file (available at http://www.metakognitivetherapie.de/) for constructing the ATT condition in the fMRI paradigm. However, written instructions were displayed on a computer screen in the scanner (see Figure 1). In addition, while high quality noise cancellation headphones (<https://www.optoacoustics.com/medical/optoactive/features>) limited the noise of the scanner, it was still be part of the audio sensation of the participant. In first pilot subjects we checked the audibility of the different sounds and removed sounds of insects and water from the instruction, leaving four different sounds in the ATT condition (clock ticking, church bell, bird song and traffic).
      2. Control condition: In line with Rosenbaum et al. (2018), we included a control condition where participants focussed passively on white noise. While this low-level control condition prevents participants to perform ATT involuntarily, it differs from the ATT condition in multiple aspects besides the AC component (e.g., the variable number, composition and volume of sounds). For example, the frequency ranges (see Fig. S1) differed between the conditions leading to differences in auditory complexity which we would like to balance between ATT and control conditions. Therefore, we included two higher-level control conditions, in which subjects listen to 1) alternative sounds or 2) the same sounds as in the ATT conditions but without the instruction to selectively focus on specific sounds. The alternative sounds (consisting of keyboard clicks, reverse driving warning sound, waves and seagulls), were created using the WavePad Audio-Editor **(**[Audiobearbeitungsprogramm. Sound, Musik, Stimmen & MP3 bearbeiten und schneiden (nch.com.au)](https://www.nch.com.au/wavepad/de/index.html?theme=mp3&kw=mp3%20schneiden%20bearbeiten&m=e&d=c&c=705598862877&ag=990915765&gad_source=1&gclid=CjwKCAjw6JS3BhBAEiwAO9waF0GGLKdJwm-bzlDhGk5TgQPLgMP5ibp0UbyCoFnVtxfEf5wZ3ldxPhoCV1MQAvD_BwE))**.**

**Figure S1: Comparison of frequency distribution between conditions paralleled with the scanner noise**


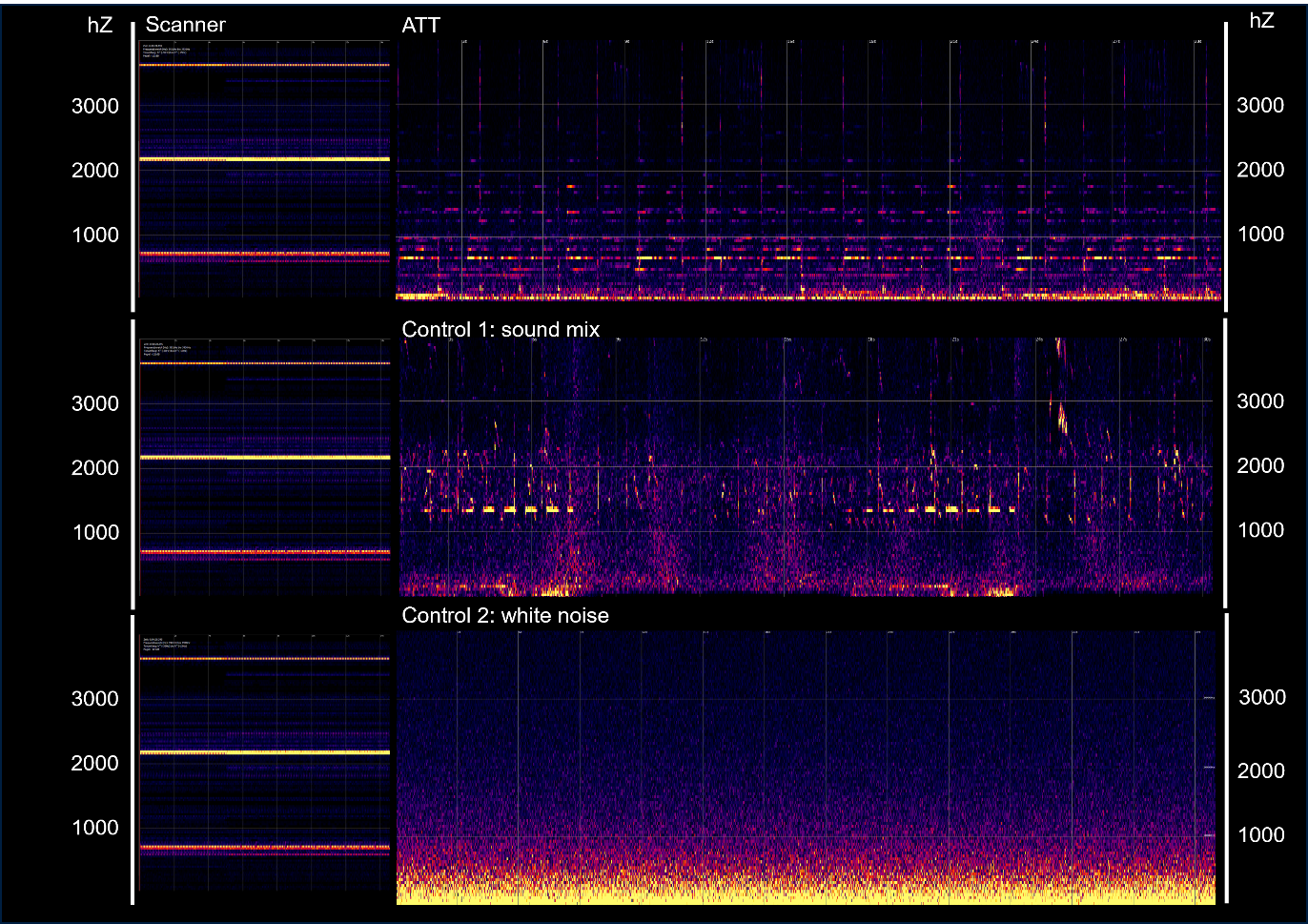


1. **Task design**

In line with Rosenbaum et al. (2018), we used a block design. The fMRI task contrasted two ATT condition (ATT_switch_, ATT_focus_) to two passive-listening control conditions, one using white noise (CON_white_) and one using alternative sounds (CON_sounds_). The task was slightly modified for testing the second independent sample. Instead of the white noise (CON_white_) we used a higher-level control condition using ATT sounds (CON_ATT_). The exact task parameters of both versions are outlined below and in Figure 1.

*Sample 1:* Each trial started with a visual instruction (ATT trial: Focus your attention [German: “Achtung, Aufmerksamkeit fokussieren”]; control trials: Listen passively [German: “Gedanken wandern lassen”]) shown for 2 seconds, followed by a 500 ms screen with a fixation cross. A 34-second sound clip was then presented. During this time, a visually presented word indicated the attentional focus in the ATT condition (e.g., bird, water [German: “Vogel”, “Wasser”) or prompted passive listening in the control conditions (e.g., listen [German: entspannt zuhören]). In the ATT_switch_ condition, the cue changed seven times every 4.8 seconds, whereas it remained constant in the other conditions. After each block, participants saw a fixation cross for 1 seconds, before they rated their current attentional direction (internal vs. external) and effort, each presented for 4 seconds (see Figure 1). After another 1-second fixation pause, the next trial began. Each trial lasted 46 seconds, and each of the four conditions was presented four times in pseudorandomized order, resulting in a total task duration of 12.4 minutes.

*Sample 2:* While the basic structure remained similar, several adaptations were made to reduce temporal predictability and enhance ecological validity. The trial again began with a 2-second instruction, followed by a jittered inter-trial interval (ITI, 0.2–2 seconds). The main sound duration was shortened to 19.5 seconds. During playback, participants again saw an attentional cue (e.g., traffic, water [German: “Verkehr”, “Wasser”] in ATT or listen [German: entspannt zuhören] in control trails). To reduce the predictability of switching points, we inserted short 500 ms visual interruptions after jittered intervals (2.5–4.5 seconds). In the ATT_switch_ condition, the cue changed after these pauses; in the other conditions, the same cue reappeared. As in Sample 1, a 1-second delay preceded the effort and attentional focus ratings (4 seconds each), followed by another 1-second delay before the start of the next trial. Each trial now lasted 32.5 seconds, and 25 trials were presented in pseudorandomized order, resulting in a slightly longer total task duration of 14 minutes.

1. **Task administration procedure**

The paradigm was presented using Presentation software (Neurobehavioral Systems, Albany, CA, USA). Before entering the scanner, participants received standardized instructions and completed a short practice session to familiarize themselves with the auditory stimuli (including both task-related sounds and scanner noise), attentional focus cues, and the rating procedure. At the beginning of the scanning session, the system volume was initially set to a medium level and gradually increased until the participant confirmed that they could clearly hear the auditory instructions and stimuli. This procedure ensured individual adaptation to scanner noise and optimized audibility through the noise-cancelling headphones. To minimize confusion during the task, participants were informed that some target sounds might be difficult to detect due to scanner noise and were encouraged to listen attentively and wait until the sounds became identifiable. For the rating procedure, they were instructed to use their index fingers to move the cursor and their left thumb to confirm responses via the *Enter* key. They were also trained to recognize the visual feedback (cursor turning red) to ensure response registration. Participants were encouraged to respond intuitively and to base their ratings on the preceding block rather than their current state.

1. **Investigated Contrasts of Interest**

**Table S1:** Task contrasts of interest in sample 1 and 2

|  | Contrast | Sample 1 | Sample 2 |
| --- | --- | --- | --- |
| Main Task Effect | ATT > CON | x | x |
| Test of ATT conditions | ATT_switch_ > CON | x | x |
|  | ATT_focus_ > CON | x | x |
|  | ATT_switch_ vs. ATT_focus_ | x | x |
| Differential effects of control conditions | ATT > CON_sound_ | x | x |
|  | ATT > CON_white_ | x |  |
|  | ATT > CON_ATT_ |  | x |
|  | CON_sound_ vs. CON_white_ | x |  |
|  | CON_sound_ vs. CON_ATT_ |  | x |

ATT: Attention Training Technique condition; CON: control condition; ATT_switch_: switch the attentional focus rapidly between sounds; ATT_focus_: focus attention to a specific sound for a longer period of time; CON_sound_: Passively listen to alternative sounds; CON_white_: Passively listen to white noise (low-level control); CON_ATT_: Passively listen to ATT sounds. Note that CON_white_ was only assessed in Sample 1, and CON_ATT_ was only assessed in Sample 2.

1. **fMRI results**

**Table S2:** Task effect (ATT > CON) in whole brain analyses

| Brain region (AAL3) | MNI coordinates | | | *T* | *k* | *p_FWE_* |
| --- | --- | --- | --- | --- | --- | --- |
|  | *x* | *y* | *z* |  |  |  |
| *sample 1* |  |  |  |  |  |  |
| R Superior Temporal Gyrus | 63 | -37 | 8 | 10.08 | 1268 | <.001 |
| L Superior Temporal Gyrus | -51 | -16 | 5 | 10.01 | 1469 | <.001 |
| R Middle Occipital Gyrus | 30 | -88 | -1 | 8.93 | 106 | <.001 |
| R Inferior Frontal Gyrus, Triangular Part | 42 | 14 | 20 | 8.32 | 913 | <.001 |
| L Middle Occipital Gyrus | -24 | -94 | -1 | 8.06 | 133 | <.001 |
| L Lobule VIIB of Cerebellar Hemisphere | -33 | -64 | -49 | 7.68 | 208 | <.001 |
| R Lobule VIII of Cerebellar Hemisphere | 27 | -67 | -49 | 7.37 | 24 | <.001 |
| L Precentral Gyrus | -36 | 2 | 62 | 6.69 | 19 | .001 |
| R Lobule VI of Cerebellar Hemisphere | 33 | -64 | -28 | 6.48 | 43 | .001 |
| R Middle Frontal Gyrus | -33 | 44 | 14 | 6.19 | 30 | .003 |
| L Inferior Frontal Gyrus, Triangular Part | -36 | 29 | 11 | 6.15 | 65 | .004 |
| R Supplementary Motor Area | 6 | 5 | 62 | 5.72 | 25 | .012 |
| *sample 2* |  |  |  |  |  |  |
| L Inferior Parietal Gyrus | -33 | -52 | 41 | 8.75 | 134 | <.001 |
| R Inferior Parietal Gyrus | 48 | -40 | 47 | 7.87 | 143 | <.001 |
| R Superior Temporal Gyrus | 57 | -37 | 11 | 7.58 | 38 | .001 |
| R Inferior Occipital Gyrus | 27 | -91 | -7 | 7.00 | 38 | .003 |
| L Superior Temporal Gyrus | -60 | -43 | 20 | 6.98 | 33 | .003 |
| R Lobule VIII of Cerebellar Hemisphere | 21 | -73 | -49 | 6.92 | 20 | .004 |
| L Inferior Occipital Gyrus | -27 | -97 | -7 | 6.89 | 21 | .004 |
| L Precentral Gyrus | -42 | 2 | 26 | 6.47 | 16 | .009 |

*Note*: Regions were classified according to the Automated Anatomical Labeling Atlas (Rolls et al., 2020). Cluster extent *k* is given at *p* < .05, familywise error (FWE) corrected for multiple comparisons within the whole brain for *k*>10 voxels. *x*-, *y*-, and *z*-coordinates (MNI) and statistical information refer to the peak voxel(s) in the corresponding cluster (voxel-level statistics). R, right; L, left.

**Table S3:** Task effect (ATT > CON) in ROI analyses

| Brain region (AAL3) | MNI coordinates | | | *T* | *k* | *p_FWE_* |
| --- | --- | --- | --- | --- | --- | --- |
|  | *x* | *y* | *z* |  |  |  |
| *sample 1* |  |  |  |  |  |  |
| R Inferior Frontal Gyrus, Triangular Part | 42 | 14 | 26 | 7.39 | 384 | <.001 |
| L Precentral Gyrus | -39 | 2 | 35 | 6.61 | 300 | <.001 |
| R Middle Frontal Gyrus | 33 | 44 | -13 | 5.44 | 16 | .001 |
| L Middle Frontal Gyrus | -30 | 50 | 8 | 4.25 | 10 | .039 |
| *sample 2* |  |  |  |  |  |  |
| L Precentral Gyrus | -42 | 2 | 26 | 6.47 | 206 | <.001 |
| R Middle Frontal Gyrus | 42 | 38 | 29 | 4.48 | 219 | .003 |

*Note*: Regions were classified according to the Automated Anatomical Labeling Atlas (Rolls et al., 2020). Cluster extent *k* is given at *p* < .05, familywise error (FWE) corrected for multiple comparisons within the predefined lPFC mask extracted from Glasser et al., (Glasser et al., 2016) for *k*>10 voxels. *x*-, *y*-, and *z*-coordinates (MNI) and statistical information refer to the peak voxel(s) in the corresponding cluster (voxel-level statistics). R, right; L, left.

**Table S4:** Comparison of ATT conditions (whole brain results)

| Brain region (AAL3) | MNI coordinates | | | | | *T* | *k* | *p_FWE_* |
| --- | --- | --- | --- | --- | --- | --- | --- | --- |
|  | *x* | | *y* | | *z* |  |  |  |
| ***ATT_switch_ > CON***  *sample 1* |  | |  | |  |  |  |  |
| L Inferior Frontal Gyrus, Triangular Part | -45 | | 14 | | 26 | 11.69 | 3023 | <.001 |
| R Middle Temporal Gyrus | 63 | | -37 | | 8 | 10.14 | 3327 | <.001 |
| L Lobule VI of Cerebellar Hemisphere | -27 | | -58 | | -34 | 9.80 | 617 | <.001 |
| L Middle Occipital Gyrus | -21 | | -94 | | -1 | 9.76 | 211 | <.001 |
| R Middle Occipital Gyrus | 27 | | -91 | | -1 | 9.41 | 143 | <.001 |
| R Lobule VI of Cerebellar Hemisphere | 30 | | -67 | | -28 | 9.36 | 193 | <.001 |
| R Lobule VIII of Cerebellar Hemisphere | 30 | | -67 | | -49 | 9.14 | 91 | <.001 |
| L Supplementary Motor Area | -6 | | 14 | | 50 | 8.13 | 256 | <.001 |
| L Lobule IV, V of Cerebellar Hemisphere | -3 | | -52 | | -10 | 6.50 | 40 | .001 |
| R Crus II of Cerebellar Hemisphere | 12 | | -76 | | -34 | 6.55 | 36 | .002 |
| R Middle Frontal Gyrus | 33 | | 47 | | -13 | 6.27 | 10 | .003 |
| *sample 2* |  | |  | |  |  |  |  |
| L Inferior Parietal Gyrus | -33 | | -55 | | 47 | 10.41 | 226 | <.001 |
| L Superior Temporal Gyrus | -60 | | -43 | | 20 | 8.80 | 75 | <.001 |
| R Superior Temporal Gyrus | 57 | | -37 | | 14 | 8.35 | 88 | <.001 |
| R Lobule VIII of Cerebellar Hemisphere | 21 | | -73 | | -49 | 7.95 | 69 | <.001 |
| R Inferior Parietal Gyrus | 48 | | -37 | | 47 | 7.86 | 157 | .001 |
| R Inferior Occipital Gyrus | 27 | | -94 | | -7 | 7.64 | 51 | .001 |
| L Insula | -30 | | 17 | | 8 | 7.62 | 40 | .001 |
| L Precentral Gyrus | -51 | | 2 | | 47 | 7.10 | 138 | .003 |
| L Middle Occipital Gyrus | -27 | | -97 | | -4 | 6.91 | 26 | .004 |
| R Insula | 30 | | 23 | | 8 | 6.86 | 36 | .004 |
| L Postcentral Gyrus | -60 | | -19 | | 26 | 6.58 | 28 | .008 |
| L Supplementary Motor Area | 0 | | 8 | | 56 | 6.33 | 34 | .013 |
| R Lobule VI of Cerebellar Hemisphere | 27 | | -61 | | -28 | 6.31 | 14 | .014 |
| R Inferior Frontal Gyrus, Triangular Part | 45 | | 32 | | 26 | 6.11 | 19 | .021 |
|  |  | |  | |  |  |  |  |
| **Table S4 (continued)** |  | | | | |  |  |  |
| Brain region (AAL3) | MNI coordinates | | | | | *T* | *k* | *p_FWE_* |
|  | *x* | *y* | | *z* | |  |  |  |
| ***ATT_focus_ > CON***  *sample 1* |  | |  | |  |  |  |  |
| L Superior Temporal Gyrus | -51 | | -16 | | 5 | 7.84 | 171 | <.001 |
| R Inferior Occipital Gyrus | 30 | | -88 | | -4 | 7.05 | 47 | <.001 |
| R Superior Temporal Gyrus | 60 | | -16 | | 5 | 6.93 | 156 | <.001 |
| L Inferior Occipital Gyrus | -27 | | -91 | | -7 | 5.75 | 18 | .011 |
| *sample 2* |  | |  | |  |  |  |  |
| R Inferior Occipital Gyrus | 30 | | -91 | | -10 | 6.51 | 18 | .018 |
| ***ATT_switch_ > ATT_focus_***  *sample 1* |  | |  | |  |  |  |  |
| L Precentral Gyrus | -42 | | -1 | | 32 | 7.95 | 401 | <.001 |
| L Inferior Parietal Gyrus | -30 | | -52 | | 41 | 6.83 | 91 | .001 |
| L Supplementary Motor Area | -6 | | 11 | | 53 | 6.79 | 95 | .001 |
| L Fusiform Gyrus | -21 | | -79 | | -10 | 6.50 | 333 | .001 |
| L Middle Temporal Gyrus | -54 | | -52 | | 11 | 6.45 | 86 | .002 |
| R Calcarine Fissure and Surrounding Cortex | 12 | | -94 | | -1 | 6.30 | 54 | .003 |
| R Middle Frontal Gyrus | 39 | | 2 | | 38 | 6.04 | 21 | .005 |
| R Insula | 27 | | 26 | | 2 | 5.88 | 16 | .008 |
| R Lobule VI of Cerebellar Hemisphere | 30 | | -61 | | -28 | 5.87 | 29 | .008 |
| R Temporal Superior Gyrus | 51 | | -40 | | 11 | 5.87 | 23 | .009 |
| R Lobule VIII of Cerebellar Hemisphere | 33 | | -64 | | -49 | 5.80 | 17 | .010 |
| L Calcarine Fissure | -9 | | -94 | | -10 | 5.74 | 12 | .013 |
| *sample 2* |  | | *No results* | |  |  |  |  |

*Note*: Regions were classified according to the Automated Anatomical Labeling Atlas (Rolls et al., 2020). Cluster extent *k* is given at *p* < .05, familywise error (FWE) corrected for multiple comparisons within the whole brain for *k*>10 voxels. *x*-, *y*-, and *z*-coordinates (MNI) and statistical information refer to the peak voxel(s) in the corresponding cluster (voxel-level statistics). R, right; L, left.

**Table S5:** Comparison of ATT conditions (ROI results)

| Brain region (AAL3) | MNI coordinates | | | *T* | *k* | *p_FWE_* |
| --- | --- | --- | --- | --- | --- | --- |
|  | *x* | *y* | *z* |  |  |  |
| ***ATT_switch_ > CON***  *sample 1* |  |  |  |  |  |  |
| L Rolandic Operculum | -42 | 2 | 14 | 9.87 | 329 | <.001 |
| R Inferior Frontal Operculum | 42 | 11 | 26 | 8.75 | 386 | <.001 |
| R Middle Frontal Gyrus | 33 | 44 | -13 | 6.03 | 16 | <.001 |
| *sample 2* |  |  |  |  |  |  |
| L Precentral Gyrus | -42 | 2 | 26 | 6.90 | 235 | <.001 |
| R Inferior Frontal Gyrus, Triangular Part | 45 | 32 | 29 | 6.00 | 258 | .001 |
| ***ATT_focus_ > CON***  *sample 1* |  |  |  |  |  |  |
| R Middle Frontal Gyrus | 42 | 26 | 38 | 4.35 | 291 | .030 |
| *sample 2* |  |  |  |  |  |  |
| L Inferior Frontal Gyrus, Triangular Part | -42 | 2 | 26 | 4.43 | 17 | .048 |
| ***ATT_switch_ > ATT_focus_***  *sample 1* |  |  |  |  |  |  |
| L Precentral Gyrus | -42 | 2 | 32 | 7.61 | 179 | <.001 |
| R Middle Frontal Gyrus | 48 | 35 | 32 | 5.89 | 186 | <.001 |
| *sample 2* |  |  |  |  |  |  |
| L Inferior Frontal Operculum | -42 | 2 | 32 | 4.71 | 135 | .024 |

*Note*: Regions were classified according to the Automated Anatomical Labeling Atlas (Rolls et al., 2020). Cluster extent *k* is given at *p* < .05, familywise error (FWE) corrected for multiple comparisons within the predefined lPFC mask extracted from Glasser et al., 2016 (Glasser et al., 2016) for *k*>10 voxels. *x*-, *y*-, and *z*-coordinates (MNI) and statistical information refer to the peak voxel(s) in the corresponding cluster (voxel-level statistics). R, right; L, left.

**Figure S2: Overlap of task activation in the ATT paradigm with large-scale brain networks.**

**
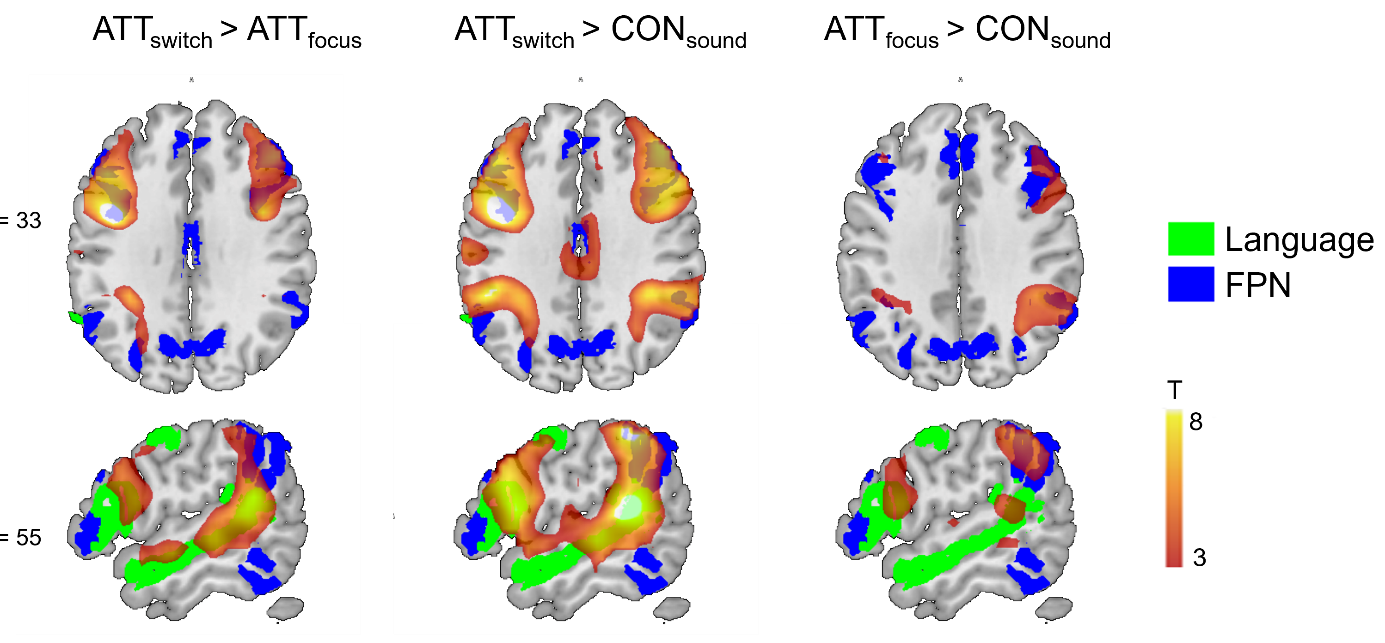
**

*Note:* Activation in main task contrasts is overlayed on large-scale brain networks derived from Glasser et al., 2016 parcellation to distinguish fronto-parietal control (FPN) from language processing networks.

**Table S6:** Comparison of CON conditions (whole brain results)

| Brain region (AAL3) | MNI coordinates | | | *T* | *k* | *p_FWE_* |
| --- | --- | --- | --- | --- | --- | --- |
|  | *x* | *y* | *z* |  |  |  |
| ***ATT > CON_sound_***  *sample 1* |  |  |  |  |  |  |
| R Middle Occipital Gyrus | 30 | -88 | -1 | 7.98 | 84 | <.001 |
| L Middle Occipital Gyrus | -21 | -94 | -1 | 7.20 | 91 | <.001 |
| R Inferior Frontal Gyrus, Triangular Part | 42 | 14 | 20 | 7.11 | 225 | <.001 |
| R Middle Temporal Gyrus | 51 | -40 | 8 | 7.05 | 464 | <.001 |
| L Inferior Frontal Gyrus, Triangular Part | -48 | 11 | 23 | 6.97 | 85 | <.001 |
| L Middle Temporal Gyrus | -48 | -46 | 20 | 6.89 | 110 | <.001 |
| L Lobule VIII of Cerebellar Hemisphere | -30 | -64 | -46 | 6.08 | 32 | .005 |
| L Lobule VI of Cerebellar Hemisphere | -27 | -64 | -28 | 6.07 | 36 | .005 |
| R Precentral Gyrus | 48 | 2 | 47 | 6.06 | 20 | .005 |
| R Lobule VIII of Cerebellar Hemisphere | 24 | -67 | -49 | 6.02 | 16 | .005 |
| Insula L | -36 | 26 | 11 | 5.82 | 11 | .007 |
| R Middle Frontal Gyrus | 42 | 38 | 26 | 5.74 | 20 | .012 |
| *sample 2* |  |  |  |  |  |  |
| L Inferior Parietal Gyrus | -33 | -49 | 41 | 8.15 | 100 | <.001 |
| R Inferior Parietal Gyrus | 39 | -46 | 41 | 5.23 | 58 | .003 |
| R Inferior Occipital Gyrus | 30 | -94 | -4 | 4.81 | 13 | .017 |
| ***ATT > CON_white_ (****sample 1)* |  |  |  |  |  |  |
| L Superior Temporal Gyrus | -51 | -25 | 5 | 12.72 | 1126 | <.001 |
| R Superior Temporal Gyrus | 57 | -16 | 2 | 11.96 | 883 | <.001 |
| R Inferior Occipital Gyrus | 30 | -91 | -4 | 8.19 | 89 | <.001 |
| R Inferior Frontal Gyrus, Opercular Part | 45 | 11 | 26 | 8.04 | 871 | <.001 |
| L Inferior Occipital Gyrus | -24 | -94 | -4 | 7.75 | 127 | <.001 |
| L Inferior Frontal Gyrus, Triangular Part | -45 | 17 | 26 | 7.65 | 329 | <.001 |
| R Inferior Parietal Gyrus | 51 | -40 | 47 | 7.63 | 348 | <.001 |
| L Lobule VIIB of Cerebellar Hemisphere | -33 | -64 | -49 | 6.80 | 43 | <.001 |
| L Precentral Gyrus | -33 | 2 | 65 | 6.39 | 16 | .001 |
| L Middle Frontal Gyrus | -33 | 44 | 14 | 6.31 | 36 | .002 |
| L Supplementary Motor Area | -6 | 8 | 59 | 6.13 | 44 | .002 |
| L Cerebellum Crus 1 | -27 | -64 | -31 | 6.08 | 46 | .004 |
| L Insula | -33 | 20 | 2 | 5.69 | 16 | .012 |
| R Cerebellum Crus 1 | 30 | -67 | -28 | 5.58 | 47 | .017 |
| ***ATT > CON_ATT_ (****sample 2)* |  |  |  |  |  |  |
| R Inferior Occipital Gyrus | 27 | -91 | -7 | 8.57 | 31 | <.001 |
| R Supra Marginal Gyrus | 51 | -43 | 44 | 8.36 | 54 | .001 |
| L Inferior Parietal Gyrus | -33 | -55 | 44 | 7.74 | 36 | .002 |
| R Lobule VIII of Cerebellar Hemisphere | 24 | -70 | -49 | 7.44 | 23 | .003 |
| L Inferior Occipital Gyrus | -27 | -97 | -7 | 7.44 | 34 | .003 |
| R Inferior Frontal Gyrus, Triangular Part | 51 | 17 | 26 | 6.47 | 11 | .021 |
| ***CON_sound_ vs. CON_white_ (****sample 1)* |  |  |  |  |  |  |
| L Superior Temporal Gyrus | -45 | -28 | 8 | 12.42 | 570 | <.001 |
| R Superior Temporal Gyrus | 57 | -19 | 5 | 10.59 | 496 | <.001 |
| ***CON_ATT_ vs. CON_sound_ (****sample 2)* | *No results* | | | | | |

*Note*: Regions were classified according to the Automated Anatomical Labeling Atlas (Rolls et al., 2020). Cluster extent *k* is given at *p* < .05, familywise error (FWE) corrected for multiple comparisons within the whole brain for *k*>10 voxels or within the predefined dlPFC mask (marked with *). *x*-, *y*-, and *z*-coordinates (MNI) and statistical information refer to the peak voxel(s) in the corresponding cluster (voxel-level statistics). R, right; L, left.

**Table S7:** Comparison of CON conditions (ROI results)

| Brain region (AAL3) | MNI coordinates | | | *T* | *k* | *p_FWE_* |
| --- | --- | --- | --- | --- | --- | --- |
|  | *x* | *y* | *z* |  |  |  |
| ***ATT > CON_sound_***  *sample 1* |  |  |  |  |  |  |
| R Middle Temporal Gyrus | 42 | 29 | 41 | 6.26 | 380 | <.001 |
| R Middle Temporal Gyrus | 36 | 47 | -14 | 5.10 | 12 | .003 |
| L Precentral Gyurs | -39 | 2 | 38 | 4.93 | 257 | .005 |
| *sample 2* |  |  |  |  |  |  |
| L Precentral Gyurs | -42 | 2 | 26 | 5.46 | 170 | .003 |
| R Middle Frontal Gyrus | 39 | 47 | 20 | 4.54 | 21 | .034 |
| R Middle Frontal Gyrus | 39 | 32 | 20 | 4.46 | 135 | .041 |
| ***ATT > CON_white_ (sample 1)*** |  |  |  |  |  |  |
| R Inferior Frontal Gyrus, Opercular Part | 42 | 11 | 26 | 7.85 | 373 | <.001 |
| L Inferior Frontal Gyrus, Opercular Part | -42 | 5 | 29 | 6.93 | 248 | <.001 |
| R Middle Frontal Gyrus | 33 | 44 | -10 | 5.37 | 17 | .001 |
| ***ATT > CON_ATT_ (sample 2)*** |  |  |  |  |  |  |
| L Precentral Gyurs | -42 | 2 | 26 | 5.82 | 69 | .001 |
| R Inferior Frontal Operculum | 48 | 17 | 29 | 5.61 | 220 | .002 |
| L Inferior Frontal Gyrus, Triangular Part | -42 | 47 | 8 | 4.96 | 82 | .012 |
| ***CON_sound_ vs. CON_white_ (sample 1)*** |  |  |  |  |  |  |
| L Inferior Frontal Gyrus, Opercular Part | 36 | 8 | 29 | 4.18 | 42 | .048 |
| ***CON_ATT_ vs. CON_sound_ (sample 2)*** | *No results* | | | | | |

*Note*: Regions were classified according to the Automated Anatomical Labeling Atlas (Rolls et al., 2020). Cluster extent *k* is given at *p* < .05, familywise error (FWE) corrected for multiple comparisons within the predefined lPFC mask extracted from Glasser et al., 2016 (Glasser et al., 2016) for *k*>10 voxels. *x*-, *y*-, and *z*-coordinates (MNI) and statistical information refer to the peak voxel(s) in the corresponding cluster (voxel-level statistics). R, right; L, left.

1. **Behavioral results**

**Table S8: Post-hoc differences of mean scale ratings between conditions**

| contrast | | MD | SE | p-values (BH-adjusted) | 95% CI interval | | Effect size (Cohens d_z_) |
| --- | --- | --- | --- | --- | --- | --- | --- |
| **EFFORT**  *Sample 1* |  |  |  |  |  |  |  |
| *ATT_switch_* | *ATT_focus_* | .194^*^ | .073 | .022 | .047 | .341 | .405 |
|  | *CON_sound_* | 1.477^*^ | .192 | <.001 | 1.089 | 1.865 | 1.173 |
|  | *CON_white_* | 1.694^*^ | .202 | <.001 | 1.286 | 2.101 | 1.279 |
| *ATT_focus_* | *CON_sound_* | 1.283^*^ | .186 | <.001 | .907 | 1.659 | 1.052 |
|  | *CON_white_* | 1.500^*^ | .196 | <.001 | 1.104 | 1.896 | 1.167 |
| *CON_sound_* | *CON_white_* | .217^*^ | .091 | .022 | .033 | .401 | .364 |
| *Sample 2* |  |  |  |  |  |  |  |
| *ATT_switch_* | *ATT_focus_* | .224 | .098 | .091 | .023 | .426 | .432 |
|  | *CON_ATT_* | 1.575* | .203 | <.001 | 1.159 | 1.991 | 1.466 |
|  | *CON_sound_* | 1.934* | .171 | <.001 | 1.582 | 2.285 | 2.137 |
| *ATT_focus_* | *CON_ATT_* | 1.351* | .190 | <.001 | .961 | 1.740 | 1.344 |
|  | *CON_sound_* | 1.709* | .151 | <.001 | 1.400 | 2.019 | 2.139 |
| *CON_ATT_* | *CON_sound_* | .358* | .094 | .001 | .166 | .551 | 0.72 |
| **SELF/EXTERNAL FOCUS**  *Sample 1* | | |  |  |  |  |  |
| *ATT_switch_* | *ATT_focus_* | .256* | .071 | .002 | .113 | .398 | .55 |
|  | *CON_sound_* | 1.698* | .142 | <.001 | 1.412 | 1.984 | 1.824 |
|  | *CON_white_* | 1.950* | .168 | <.001 | 1.611 | 2.288 | 1.77 |
| *ATT_focus_* | *CON_sound_* | 1.442* | .126 | <.001 | 1.188 | 1.696 | 1.745 |
|  | *CON_white_* | 1.694* | .153 | <.001 | 1.384 | 2.003 | 1.688 |
| *CON_sound_* | *CON_white_* | .252* | .103 | .019 | .043 | .461 | .373 |
| *Sample 2* |  |  |  |  |  |  |  |
| *ATT_switch_* | *ATT_focus_* | .168 | .077 | .074 | .011 | .326 | .412 |
|  | *CON_ATT_* | 1.341* | .233 | <.001 | .863 | 1.818 | 1.088 |
|  | *CON_sound_* | 1.531* | .231 | <.001 | 1.056 | 2.005 | 1.253 |
| *ATT_focus_* | *CON_ATT_* | 1.172* | .225 | <.001 | .711 | 1.633 | 0.984 |
|  | *CON_sound_* | 1.362* | .224 | <.001 | .903 | 1.821 | 1.149 |
| *CON_ATT_* | *CON_sound_* | .190 | .162 | .251 | -.142 | .522 | .222 |

Note: MD: mean difference, SE: standard error, BH: Bonferroni-Holm adjusted, CI: confidence interval.

1. **Parametric Modulation**

**Table S9: Whole brain Parametric Modulation Analyses**

| Brain region (AAL3) | MNI coordinates | | | *T* | *k* | *p_FWE_* |
| --- | --- | --- | --- | --- | --- | --- |
|  | *x* | *y* | *z* |  |  |  |
| **EFFORT**  *sample 1* |  |  |  |  |  |  |
| R Middle Occipital Gyrus | 39 | -85 | -10 | 7.04 | 88 | <.011 |
| R Superior Temporal Gyrus | 66 | -37 | 8 | 6.96 | 278 | <.001 |
| L Middle Occipital Gyrus | -27 | -97 | 2 | 6.76 | 71 | .001 |
| L Superior Temporal Gyrus | -45 | -34 | 5 | 6.21 | 95 | .001 |
| L Superior Temporal Gyrus | -39 | -43 | 20 | 6.28 | 11 | .002 |
| L Precentral Gyrus | -48 | 5 | 50 | 6.27 | 52 | .002 |
| L Superior Temporal Gyrus | -63 | -40 | 11 | 6.21 | 45 | .003 |
| L Inferior Frontal Gyrus, Opercular Part | -39 | 5 | 29 | 5.93 | 22 | .006 |
| R Inferior Parietal Gyrus | 51 | -46 | 53 | 5.80 | 19 | .009 |
| R Middle Frontal Gyrus | 39 | 29 | 29 | 5.79 | 29 | .009 |
| L Supra Marginal | -45 | -43 | 32 | 5.73 | 16 | .011 |
| *sample 2* |  |  |  |  |  |  |
| L Inferior Parietal Gyrus | -33 | -55 | 44 | 8.15 | 36 | <.001 |
| R Inferior Parietal Gyrus | 36 | -49 | 41 | 7.17 | 24 | .002 |
| **EXTERNAL > SELF-FOCUS**  *sample 1* | | |  |  |  |  |
| R Superior Temporal Gyrus | 63 | -40 | 14 | 9.48 | 830 | <.001 |
| L Precentral Gyrus | -39 | 2 | 29 | 8.38 | 431 | <.001 |
| L Inferior Parietal Gyrus | -48 | -40 | 38 | 8.01 | 549 | <.001 |
| R Inferior frontal gyrus, Triangular Part | 51 | 17 | 23 | 7.60 | 389 | <.001 |
| R Lobule VIII of Cerebellar Hemisphere | -36 | -61 | -49 | 7.06 | 158 | <.001 |
| L Inferior Occipital Gyrus | -24 | -94 | -4 | 6.88 | 85 | <.001 |
| R Lobule VIII of Cerebellar Hemisphere | 27 | -70 | -49 | 6.67 | 26 | .001 |
| L Inferior Occipital Gyrus | 24 | -94 | -7 | 6.47 | 39 | .002 |
| R Crus I of Cerebellar Hemisphere | 36 | -64 | -28 | 6.03 | 21 | .005 |
| R Precentral Gyrus | 42 | 2 | 47 | 5.98 | 21 | .006 |
| R Insula | 36 | 20 | 8 | 5.60 | 15 | .018 |
| *sample 2* |  |  |  |  |  |  |
| L Superior Temporal Gyrus | -57 | -43 | 14 | 8.16 | 49 | <.001 |
| R Superior Temporal Gyrus | 60 | -37 | 11 | 6.53 | 43 | .008 |
| R Middle Frontal Gyrus | 48 | 23 | 23 | 5.42 | 238 | .005 |
| L Inferior Frontal Gyrus, Opercular Part | -39 | 11 | 29 | 5.01 | 170 | .013 |

*Note*: Regions were classified according to the Automated Anatomical Labeling Atlas (Rolls et al., 2020). Cluster extent *k* is given at *p* < .05, familywise error (FWE) corrected for multiple comparisons within the whole brain for *k*>10 voxels. *x*-, *y*-, and *z*-coordinates (MNI) and statistical information refer to the peak voxel(s) in the corresponding cluster (voxel-level statistics). R, right; L, left.

**Table S10: ROI Parametric Modulation Analyses**

| Brain region (AAL3) | MNI coordinates | | | *T* | *k* | *p_FWE_* |
| --- | --- | --- | --- | --- | --- | --- |
|  | *x* | *y* | *z* |  |  |  |
| **EFFORT**  *sample 1* |  |  |  |  |  |  |
| R Middle Occipital Gyrus | 39 | -85 | -10 | 7.04 | 88 | <.011 |
| R Superior Temporal Gyrus | 66 | -37 | 8 | 6.96 | 278 | <.001 |
| L Middle Occipital Gyrus | -27 | -97 | 2 | 6.76 | 71 | .001 |
| L Superior Temporal Gyrus | -45 | -34 | 5 | 6.21 | 95 | .001 |
| L Superior Temporal Gyrus | -39 | -43 | 20 | 6.28 | 11 | .002 |
| L Precentral Gyrus | -48 | 5 | 50 | 6.27 | 52 | .002 |
| L Superior Temporal Gyrus | -63 | -40 | 11 | 6.21 | 45 | .003 |
| L Inferior Frontal Gyrus, Opercular Part | -39 | 5 | 29 | 5.93 | 22 | .006 |
| R Inferior Parietal Gyrus | 51 | -46 | 53 | 5.80 | 19 | .009 |
| R Middle Frontal Gyrus | 39 | 29 | 29 | 5.79 | 29 | .009 |
| L Supra Marginal | -45 | -43 | 32 | 5.73 | 16 | .011 |
| *sample 2* |  |  |  |  |  |  |
| L Inferior Parietal Gyrus | -33 | -55 | 44 | 8.15 | 36 | <.001 |
| R Inferior Parietal Gyrus | 36 | -49 | 41 | 7.17 | 24 | .002 |
| **EXTERNAL > SELF-FOCUS**  *sample 1* | | |  |  |  |  |
| R Superior Temporal Gyrus | 63 | -40 | 14 | 9.48 | 830 | <.001 |
| L Precentral Gyrus | -39 | 2 | 29 | 8.38 | 431 | <.001 |
| L Inferior Parietal Gyrus | -48 | -40 | 38 | 8.01 | 549 | <.001 |
| R Inferior frontal gyrus, Triangular Part | 51 | 17 | 23 | 7.60 | 389 | <.001 |
| R Lobule VIII of Cerebellar Hemisphere | -36 | -61 | -49 | 7.06 | 158 | <.001 |
| L Inferior Occipital Gyrus | -24 | -94 | -4 | 6.88 | 85 | <.001 |
| R Lobule VIII of Cerebellar Hemisphere | 27 | -70 | -49 | 6.67 | 26 | .001 |
| L Inferior Occipital Gyrus | 24 | -94 | -7 | 6.47 | 39 | .002 |
| R Crus I of Cerebellar Hemisphere | 36 | -64 | -28 | 6.03 | 21 | .005 |
| R Precentral Gyrus | 42 | 2 | 47 | 5.98 | 21 | .006 |
| R Insula | 36 | 20 | 8 | 5.60 | 15 | .018 |
| *sample 2* |  |  |  |  |  |  |
| L Superior Temporal Gyrus | -57 | -43 | 14 | 8.16 | 49 | <.001 |
| R Superior Temporal Gyrus | 60 | -37 | 11 | 6.53 | 43 | .008 |
| R Middle Frontal Gyrus | 48 | 23 | 23 | 5.42 | 238 | .005 |
| L Inferior Frontal Gyrus, Opercular Part | -39 | 11 | 29 | 5.01 | 170 | .013 |

*Note*: Regions were classified according to the Automated Anatomical Labeling Atlas (Rolls et al., 2020). Cluster extent *k* is given at *p* < .05, familywise error (FWE) corrected for multiple comparisons within the predefined lPFC mask extracted from Glasser et al., 2016 (Glasser et al., 2016) for *k*>10 voxels. *x*-, *y*-, and *z*-coordinates (MNI) and statistical information refer to the peak voxel(s) in the corresponding cluster (voxel-level statistics). R, right; L, left.

**Associations between FPN activation and self-reported AC**

**Table S11:** Associations between neural responses in the ATT task (ATT > CONsound) and self-reported AC

| Brain region (AAL3) | MNI coordinates | | | *T* | *k* | *p_FWE_* |
| --- | --- | --- | --- | --- | --- | --- |
|  | *x* | *y* | *z* |  |  |  |
| L Inferior Frontal Gyrus | -42 | 29 | 23 | 4.15 | 43 | .032 |

*Note*: Regions were classified according to the Automated Anatomical Labeling Atlas (Rolls et al., 2020). Cluster extent *k* is given at *p* < .05, familywise error (FWE) corrected for multiple comparisons within the predefined lPFC mask extracted from Glasser et al., 2016 (Glasser et al., 2016) for *k*>10 voxels. *x*-, *y*-, and *z*-coordinates (MNI) and statistical information refer to the peak voxel(s) in the corresponding cluster (voxel-level statistics). R, right; L, left.

**References**

Glasser, M. F., Coalson, T. S., Robinson, E. C., Hacker, C. D., Harwell, J., Yacoub, E., Ugurbil, K., Andersson, J., Beckmann, C. F., Jenkinson, M., Smith, S. M., & Van Essen, D. C. (2016). A multi-modal parcellation of human cerebral cortex. *Nature 2016 536:7615*, *536*(7615), 171–178. https://doi.org/10.1038/nature18933

Rolls, E. T., Huang, C. C., Lin, C. P., Feng, J., & Joliot, M. (2020). Automated anatomical labelling atlas 3. *NeuroImage*, *206*, 116189. https://doi.org/10.1016/J.NEUROIMAGE.2019.116189

Rosenbaum, D., Maier, M. J., Hudak, J., Metzger, F. G., Wells, A., Fallgatter, A. J., & Ehlis, A. C. (2018). Neurophysiological correlates of the attention training technique: A component study. *NeuroImage: Clinical*, *19*, 1018–1024. https://doi.org/10.1016/J.NICL.2018.06.021
